# P02 To dip or not to dip? Improving the management of lower urinary tract infections in older patients

**DOI:** 10.1093/jacamr/dlac004.001

**Published:** 2022-02-16

**Authors:** G. Muir

**Affiliations:** Senior House Officer at the Queen Elizabeth Hospital Gateshead NHS Foundation Trust, UK

## Abstract

**Introduction:**

PHE guidelines were revised in 2018 to state that urine dipsticks tests (UDTs) should *not* be used as a diagnostic aid in any patient over the age of 65 years.

The high prevalence of asymptomatic bacteriuria in the elderly population is widely acknowledged however the adherence to the above guidelines is unclear.

My aim was to investigate whether geriatric patients are being misdiagnosed with urinary tract infections (UTIs) and mistreated with unnecessary antibiotics, given the high prevalence of asymptomatic bacteriuria in this population.

**Methods:**

I sampled patients diagnosed with a UTI in the Emergency Department of the Queen Elizabeth Hospital, Gateshead over a 7 day period. I extracted medical notes of patients prescribed one of 7 antibiotics, then excluded patients under 65 years old and those treated for an infection outside the lower urinary tract. Next, I established whether a UDT was used as a diagnostic aid using the medical notes. Using this methodology, I repeated my data collection and completed two PDSA cycles of this Quality Improvement project, from 2019 to 2021.

**Results:**

My initial findings revealed that a UDT was performed in 100% patients diagnosed with a UTI (i.e. *no* cases were in line with best practice). My first educational intervention involved sharing the revised PHE guidelines with staff via an interactive teaching-session. Following this step, I found that a UDT was performed in 62% patients. My next intervention was a flow chart entitled ‘to dip or not to dip’ pasted in the ED. After this second intervention, I found that 27% cases were in line with best practice (a UDT was used in 73% cases).

**Conclusions:**

With this modest improvement, I concluded that my recommendations reduced the number of over 65-year-old patients undergoing unnecessary UDTs as part of their diagnostic work-up.

**Figure 1.**
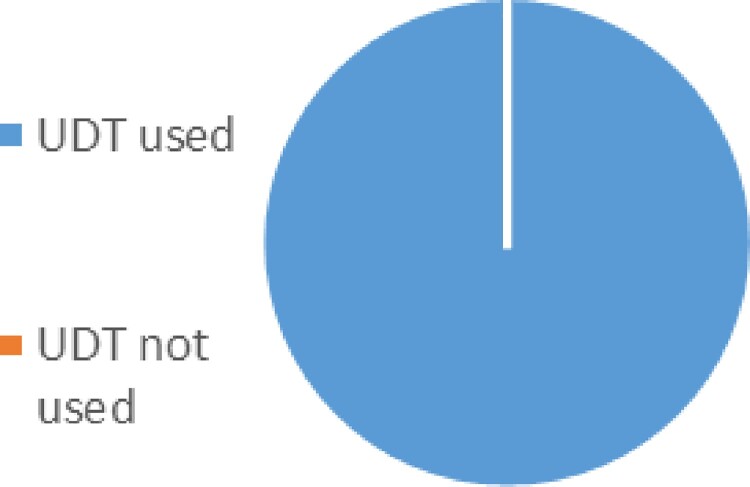
Initial results in November 2019: in 100% cases a UDT was used to diagnose a lower UTI: 0% cases in line with best practice.

**Figure 2.**
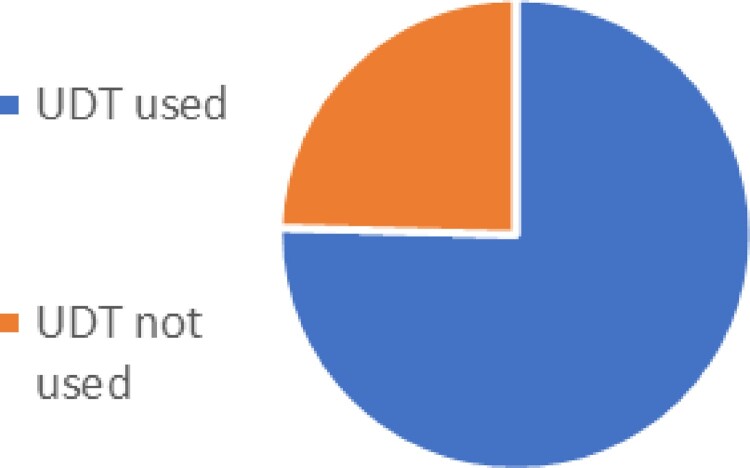
Repeat results in May 2021: in 83% cases a UDT was used to diagnose a lower UTI: 27% cases in line with best practice.

